# Structure and Dynamics of Porin Loops in Asymmetric Outer Membranes of *E. coli*

**DOI:** 10.1002/chem.71575

**Published:** 2026-08-14

**Authors:** Morgane Adélaïde, Jayasubba Reddy Yarava, Florian Lindemann, Massilia Abbas, Nils Cremer, Lisa Gerland, Matthias Hiller, Barth van Rossum, Dirk Linke, Alexander B. Barnes, Edward P. Saliba, Özkan Yildiz, Werner Kühlbrandt, Hartmut Oschkinat

**Affiliations:** ^1^ Leibniz‐ Forschungsinstitut für Molekulare Pharmakologie Berlin Germany; ^2^ Department of Biosciences University of Oslo Oslo Norway; ^3^ ETH Zürich, Lab. For Physical Chemistry Zurich Switzerland; ^4^ Ohio University Department of Chemistry and Biochemistry, 133 University Terrace Athens Ohio USA; ^5^ Max‐Planck‐Institut Für Biophysik Frankfurt am Main Germany

**Keywords:** magic angle spinning, membrane protein structure, NMR, outer membrane proteins

## Abstract

Biological membranes are usually asymmetric lipid bilayers that possess an oligosaccharide coat on the outside into which loops of transmembrane proteins extend. Structurally, the protein‐bilayer interface is not well explored. As a paradigmatic example, we examine the structure of the bacterial outer membrane protein G (OmpG) expressed into *E. coli* outer membranes (OM). We investigate two strands of the β‐sheet and loops 6 and 7 that extend into the lipopolysaccharide (LPS) layer and which are involved in pore opening (pH > 6.0) and closing (pH < 6.0). Previous x‐ray crystallography studies described these loops as well‐ordered, whereas solid‐state NMR described them as fully disordered, largely due to a lack of signal. Here, solid‐state NMR measurements of OmpG in OM vesicles at 1.2 GHz ^1^H frequency now yield extended sets of cross peaks for His and Trp residues. Two alternative conformations are observed for two His residues, which are likely H231 and H261. Trp sidechain cross peak intensities in cross polarization spectra are in agreement with an increase of conformational freedom toward the surface. The oligosaccharide layer appears to protect the loops while promoting flexibility and multiple conformations. Our investigation highlights the importance of structural investigations in a quasi‐native environment.

## Graphical abstract

What are loops of membrane proteins doing within the oligosaccharide coat of cells? This structurally unexplored space is studied by solid‐state NMR, with the outer membrane protein G (OmpG) embedded in asymmetric E. coli outer membrane (OM) vesicles as a paradigmatic example. Two conformations are observed for loops 6 and 7 at pH 7.4, relevant for switching between open and closed forms.

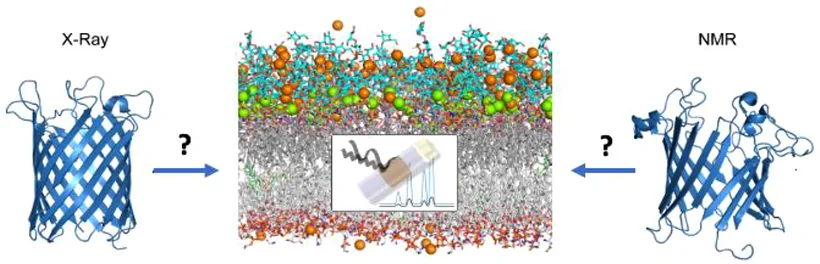

## 1 Introduction

Transmembrane β‐barrel proteins [1] on the surface of Gram‐negative bacteria, mitochondria and chloroplasts are associated with a range of different functions. They can work as enzymes, transporters or receptors [2]. The largest subfamily of OM β‐barrel proteins are porins. In the OM of Gram‐negative bacteria, they mediate the passive but often selective uptake of nutrients. OM porins are characterized by short beta turns on the periplasmic side that define the fold and structure of the β‐barrel pore. The longer loops connecting beta strands on the extracellular side often play a role in pore gating, ion or nutrient selectivity. Extracellular loops can be flexible, adopt alternative conformations or interact with lipid head groups. A realistic picture of loop structure and dynamics therefore requires a quasi‐native membrane environment.

The lipid composition of the two membrane bilayer leaflets is often different. Re‐creating this asymmetry in vitro is a major obstacle for obtaining an authentic picture of membrane protein structure and dynamics. A striking example of membrane asymmetry is the OM of Gram‐negative bacteria (Figure 1a) [3]. The outer leaflet of this membrane consists of lipopolysaccharides (LPS), while the inner leaflet is mostly composed of phospholipids [4, 5, 6]. This asymmetry extends to the hydrophobic bilayer core, with a pronounced variation of fatty acid chain length in the outer leaflet [2]. Charged groups, in particular sulphate and phosphate groups in the LPS carbohydrate surface layer coordinate divalent cations. Tight hydrogen bonding networks further contribute to rigidity and gel‐like properties of the outer layer.

**FIGURE 1:**
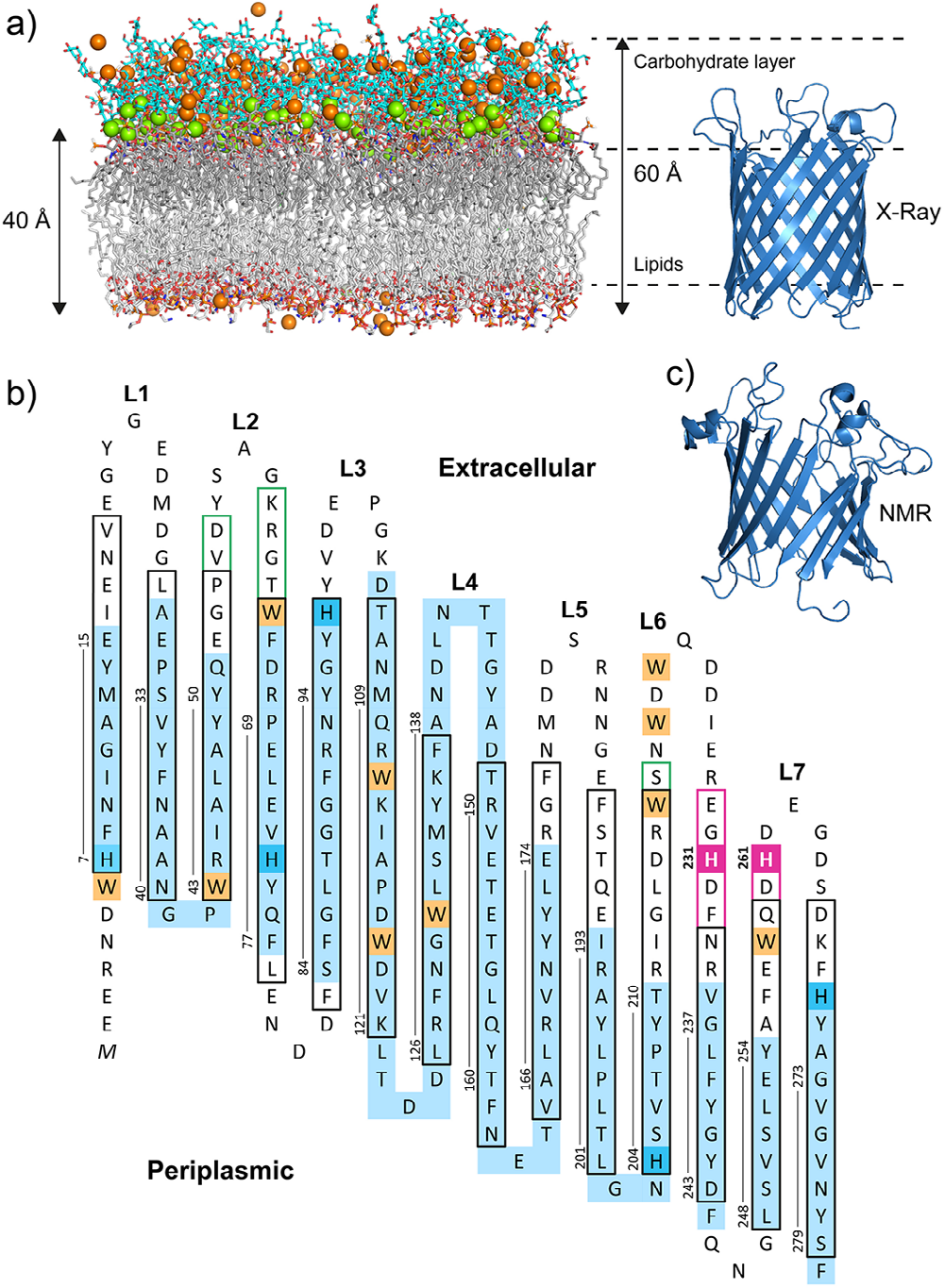
(a) Atomic representation of the Gram‐negative bacterial OM [3] as in *E. coli* BL21 (DE3), which lacks the O‐antigen. The inner leaflet consists mostly of phospholipids, while the outer leaflet is formed of LPSs. In the figure, phospholipids are light grey; lipid A is dark grey, the core region is cyan; magnesium is green, and potassium is orange. The membrane thickness measures approximately 40 Å, including all of the inner leaflet and lipid A, but up to 60 Å when the core region of the LPS is included. For comparison, the x‐ray structure of OmpG in the closed form (PDB: 2IWW, dark‐blue) is shown on the right. (b) Secondary structure of OmpG. Light blue background indicates assignments from solid‐state NMR (ssNMR). The black frame marks the extent of ß‐structure in the closed form (PDB: 2IWW), residues framed in green, magenta, or black define together the ß‐structure in the open form of OmpG (PDB: 2IWV). 10 Trp and 5 His residues are highlighted in orange and blue, respectively. H231 and H261 in β12/loop6 and β13/loop7, respectively, are highlighted by magenta background. The amino acid stretches extending β12 and β13 as discussed below and highlighted in Figure 2 are also framed in magenta. (c) Structure of OmpG determined by ssNMR (PDB: 5MWV). All structures are drawn to scale in PyMOL [7].

The thickness of the LPS carbohydrate layer can exceed that of the actual lipid bilayer, depending on the number of O‐antigen units. It is worth noting that several bacterial species and especially laboratory strains such as *E.coli* BL21 (DE3) completely lack O‐antigen in the outer leaflet, resulting in a thin but well‐defined carbohydrate layer consisting of Lipid A and core unit oligosaccharides [8].

In this work, we study the effect of the asymmetric OM on the structure and dynamics of the 34 kDa *E. coli* outer membrane protein G (OmpG) [9, 10] as a model system. We produce vesicular sample preparations derived from the outer membrane (OM) using pressure‐based cell lysis combined with selective detergent treatment to remove the inner membrane. This procedure retains all key features of the OM and its lipid asymmetry [11]. We have chosen OmpG because it is readily overexpressed in *E.coli*, and because structural studies by x‐ray crystallography [12, 13, 14] and NMR [15, 16] in detergent and various lipid environments have been published. Detailed structural information is relevant for the design of OmpG‐based nanopores for protein and DNA biosensing [17, 18, 19, 20, 21, 22]. Figure 1a indicates that the long OmpG loops may reach into the LPS core region, and their structure should in this case be sensitive to the surrounding oligosaccharides and their phosphorylation pattern. These loops are of special interest since they are thought to be involved in pH‐dependent regulation of pore activity; the channel closes when the pH drops below 6.0 [23]. X‐ray studies at different pH suggested that loop rearrangements are a prerequisite for pore opening and closing. In particular, a crystal structure obtained at pH 5.6 (PDB: 2IWW, Figure 2a) shows the closed conformation, with loop 6 covering the pore. A structure at pH 7.9 is in an open conformation where the loop 6‐lid has moved (PDB: 2IWV), and β12 and β13 are elongated (Figure 2b). The extent of secondary structure observed in the closed form (PDB: 2IWW) is indicated by black frames in Figure 1b, whereas for the open form (PDB: 2IWV), it is indicated collectively by the black, magenta and green frames. In the open form, the side chains of histidines H231 and H261 interact via a hydrogen bond between the ring nitrogens. In Figures 1b and 2a,b the amino acid stretches of β12 and β13 that are undergoing structural changes are displayed in magenta. Since β12 and β13 of the open form (Figure 2b) are longer than ln the closed form and reach into the core region of the LPS (Figure 1b, black frames, and Figure 1a, right), they are prone to being more sensitive to the LPS environment. Notably, the two x‐ray structures have different ranges of B‐factors for the loop residues. Figure 2b,c are colored according the B‐factors, the scale bar to the right is valid for both.

**FIGURE 2:**
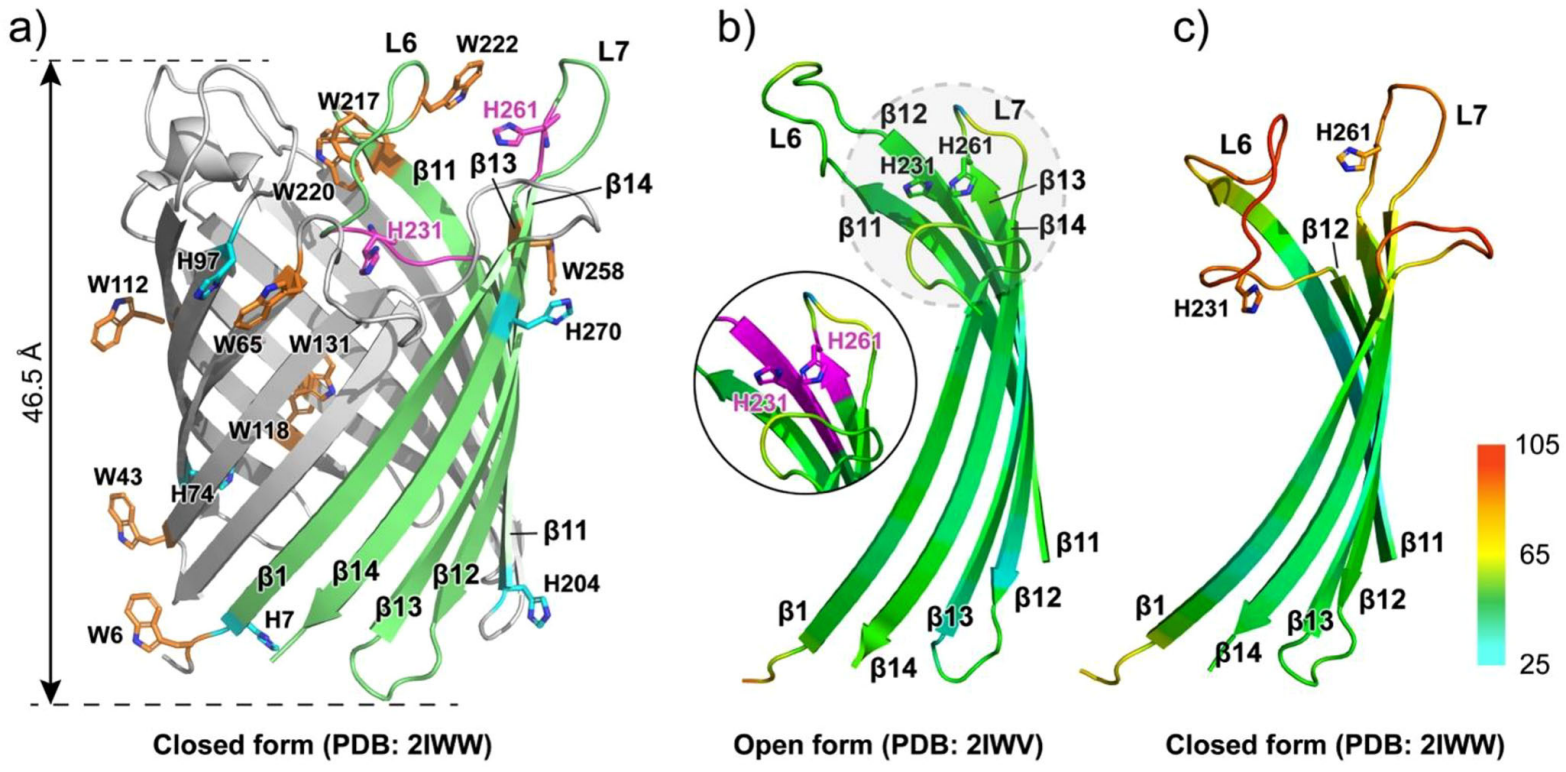
(a) X‐ray structure of OmpG at pH 5.6 in the closed state (PDB: 2IWW). The position of tryptophan residues is indicated by their side chains displayed in orange. Histidine residues are shown in cyan. The stretches colored magenta contain those residues that form the extension of β12 and β13 in the open form (see b and Figure 1b). Loops 6 and 7 are highlighted as L6 and L7, respectively. The green‐colored strands are displayed in b and c. (b) Strands β11 – β14 and β1 of the structure of the open form (PDB: 2IWV) obtained at pH 7.9, displayed with coloring according to the B‐factors of the individual residues. For the scale see right edge of the Figure. The residues that are colored magenta in a) are displayed in the insert. (c) For comparison, the five strands of the closed form colored according to the crystallographic B‐factors. They range from 25 (cyan) to 105 (red). Note the difference in L6 and L7 B‐factors between (b) and (c).

To assess the influence of the native LPS environment on the OmpG loops while reducing background signals from endogenous proteins, we expressed OmpG into the OM of a BL21 strain that lacks other major OM porins, such as OmpA, OmpC, OmpF, and LamB [24]. We isolated the OM fraction and investigated it directly by magic angle spinning (MAS) ssNMR at pH 7.4 at which the open conformation and the extended ß‐sheet conformation should be prevalent. Our experiments include detection of proton signals from [^13^C,^15^N]‐labelled samples at 100 kHz MAS and 1.2 GHz nominal ^1^H frequency, combined with amino acid type‐selective labeling of Trp and His residues. Investigation of a fully deuterated, NH re‐exchanged sample indicates that surprisingly a few NH groups of Trp side chains are solvent‐accessible. Different dynamics regimes are examined [25] by comparing spectra obtained with Hartmann‐Hahn Cross Polarization (CP) [26] and Insensitive Nuclei Enhanced by Polarization Transfer (INEPT) [27, 28].

## 2 Results and Discussion

### 2.1 Sample Preparation and Initial NMR Characterization

OmpG was expressed into the OM of *E.coli* strain BL21 *ΔABCF* lacking the genes *ompA, ompC, ompF*, and *lamB* [24]. Cells were broken and the membrane fraction was isolated. The inner membrane was removed by a light detergent wash [29], leaving an OM fraction that forms vesicles with similar properties and composition compared to naturally formed OM vesicles [11]. The vesicles investigated here are thus similar to secreted vesicles, but differ in their origin. Similar procedures with comparable bacterial strains have been shown to give good yields of target protein in native‐like environments with low background in NMR experiments [30]. For the following NMR experiments we added protease inhibitors to prevent degradation by endogenous proteases associated with the OM.

The resulting OM vesicle fraction was investigated with MAS ssNMR at pH 7.4. The ^1^H‐^15^N correlation spectrum of the open form obtained at 1.2 GHz nominal ^1^H‐frequency (Figure 3a) contains characteristic signal patterns outside the bulk amide responses (see assignments) that were previously observed in spectra of OmpG reconstituted into bilayers of LPS‐deficient *E. coli* lipids at pH 6.3 [16] (Figure 3b, dark blue spectrum). Since the two ^1^H‐^15^N correlation spectra compared in Figure 3b were recorded on samples of OmpG in very different lipid environments, and the OM vesicle sample is more heterogeneous, chemical shift changes of characteristic signals and peak duplication are also observed. Examples are the cross peaks around 119/10.2 ppm that we tentatively assign to S133 (Figure 2a). Additional cross peaks are observed in the ^1^H‐region between 9.0 and 11.0 ppm (Figure 3b, rectangle A, arrows 1 and 2) where the Trp side chain signals are usually located. Furthermore, several cross peaks that were weaker in the spectrum of OmpG in reconstituted bilayers became much more pronounced in the spectrum of OmpG in the asymmetric OM vesicles, see arrows 2 and 3, Figure 3b, as examples. Arrows 3 and white circles in Figure 3b indicate cross peaks that are characteristic of residues within ß‐sheet structures according to their inherent chemical shifts. There is also a substantial increase of cross peak intensity in the region indicated by rectangle B in Figure 3b. The additional signals in the area where Trp side chain cross peaks occur are also observed in a CP‐based ^1^H‐^15^N correlation spectrum of the same sample recorded at 900 MHz ^1^H frequency and in a spectrum recorded on an OM vesicle sample that contains OmpG selectively labeled with ^13^C,^15^N‐Trp in OM at 600 MHz (Figure S1, see also Figure S2). We attribute the slightly different positions of these cross peaks in two spectra recorded on the same sample (Figure S1a,b) to mechanical changes upon compression of the sample by MAS during the long acquisition period. 1D ^1^H spectra were recorded before and after the acquisition of 2D spectra (Figure S3), and from comparing these it can be ensured that water is not lost during the experiment. The 1D ^1^H NMR spectrum in Figure S3b (900 MHz) was recorded after weeks of acquisition on the 1.2 GHz and 900 MHz spectrometers, whereby the ratio of water peak to the background signals of the LPS sugar moieties (p1/p2) changed from 21 to 19. The spectrum of the selectively labeled sample in Figure S1c may be slightly different for other reasons, such as differences in OM composition due to altered growth conditions in different media, or the physiological state of bacteria before harvest. Regarding signal‐to‐noise (S/N), the traces were taken from the spectrum of Figure 3a which are shown in Figure S4 and yield S/N values around three for the nonsplit cross peaks, and of around 2.3 for the two components of G90 and one of S133, with the other S133 value below 2. The cross peaks emerge clearly above the noise level in Figure 3a, are well‐shaped, and appear in positions where cross peaks have been observed in the earlier study.

**FIGURE 3:**
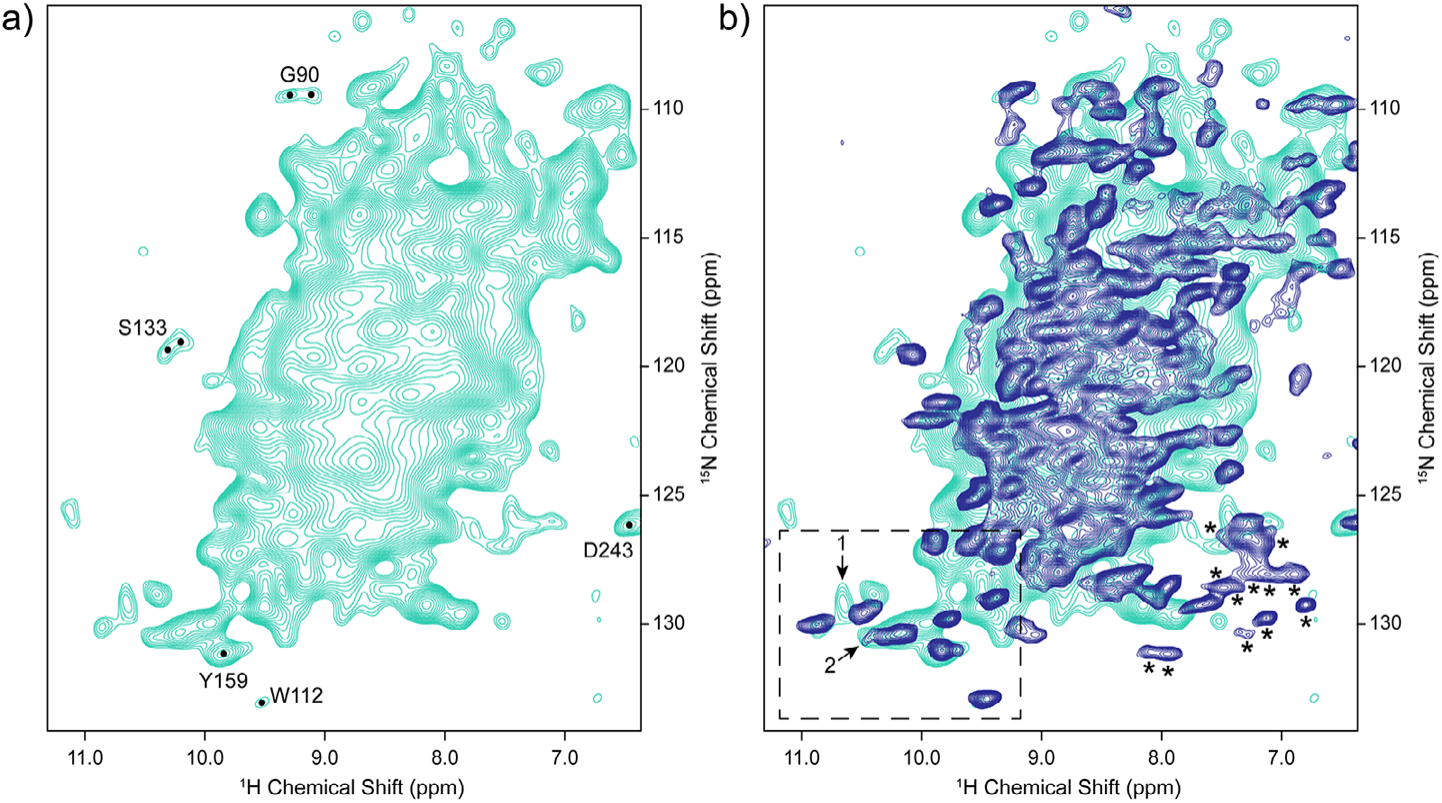
2D hNH NMR fingerprint spectra of ^13^C,^15^N‐labelled OmpG recorded at 100 kHz MAS. (a) ^1^H‐^15^N correlation by cross polarization of OmpG in OM vesicles at 1.2 GHz and pH 7.4 (turquoise). (b) Comparison of the spectrum shown in (a) with a spectrum of OmpG reconstituted into a bilayer of LPS‐deficient *E.coli* lipids recorded at 900 MHz and pH 6.3 (dark blue). Asterisks denote folded arginine side chain signals. The dashed rectangle A indicates the characteristic area of Trp side chain cross peaks. The two fused cross peaks at ^1^H/^15^N chemical shifts of 10.7/129.2 ppm indicated by arrow 1 appear only in the spectrum of the OM vesicle sample and the peak indicated by arrow 2 (10.4/130.5 ppm) is more pronounced there. Similarly, there are enhanced cross peaks at 9.5 ppm (arrows 3), and two additional cross peaks labelled with circles between 9.3 and 9.8 ppm, indicating β‐structure. Rectangle B indicates additional cross peaks in the chemical shift area that is usually typical for residues in helices and loops, without excluding other possibilities.

In summary, isolating *E. coli* OM into which we have expressed OmpG, we obtain a CP‐based ^1^H‐^15^N correlation spectrum that has all features of the same correlation obtained on purified protein incorporated into lipid bilayers—and more. In our previous investigation, a three‐dimensional (3D) HNCA experiment yielded 182 of 271 expected cross peaks involving chemical shifts of backbone atoms [15]. Furthermore, the spectral region with histidine sidechain ^13^C‐^13^C correlations showed only four sets of cross peaks of similar size at pH 4.7 or 7.5, and we observed five instead of ten Trp side chain ^1^H‐^15^N cross peaks, which we could identify by comparison with the NCA spectrum. Here, we detected additional signals in the region of Trp side chain cross peaks, and additional backbone signals. Potentially, there are different effects that may cause the appearance of the additional signals. The most obvious explanation is that we now observe signals that previously were missing due to unfavorable dynamics. Other possible explanations are that peak doubling occurs because of exchange between two conformations, heterogeneity in the membrane environment due to a varying presence of metals, or because of tight binding of different lipid molecules. An example for the latter two cases might be the splitting of S133.

### 2.2 Loop Dynamics

There are several strategies for obtaining site‐specific information on individual residues in a protein. In the ideal case an assignment procedure will yield the chemical shifts of most protein NMR signals in a residue‐specific manner, at least of all backbone atom signals. However, the signal‐to‐noise ratio of the 2D spectra obtained on our OM vesicle samples was low which makes a full, residue‐specific assignment difficult (see Table S2 for measurement times) and therefore we used an alternative approach. Considering the small number of His and Trp residues, we investigated samples with 13C, 15N‐labeled His or Trp and defined residue categories depending on cross peak intensities in CP‐ and INEPT‐based ^1^H‐^15^N or ^1^H‐^13^C correlations (for overview plots see Figure S5). In accordance with the work by Aebischer et al., we correlate maximum CP intensity with high rigidity when comparing cross peaks of the same moieties, for example, Trp side chains. The detected intensity and chemical shift patterns are correlated to the structural features and dynamics observed in the NMR and x‐ray structures, and to the signal patterns observed in spectra of our previous study. In our previous NMR investigations, large cross peaks for the blue shaded area of Figure 1b were detected by CP methods, which enabled us to obtain a sequential assignment. Backbone atoms of W43, W112, W118 and W131 were part of this assignment, as well as those of H7, H74 and H205. We further compare our data to x‐ray structures deposited in the PDB. There are four x‐ray structures (PDB: 2IWW, 2F1C, 2WVP, 7Q5C) obtained in a pH range between 5.5 and 5.75, and three at neutral to basic pH, 7.0, 7.9 and 8.5 (PDB: 2 × 9K, 2IWV, 4CTD). The pH 7.0 structure (PDB: 2 × 9K) carries the mutations H231A and H261A, and in the pH 8.5 structure (PDB: 4CTD) loop 6 had been removed. Hence, PDB: 2IWV is the only wildtype structure at basic pH. In this x‐ray structure obtained at pH 7.9, β12 and β13 are elongated, and the residues involved in the lid of the pore have moved. The previous NMR structures (PDB: 2JQY, 5MWW) are all obtained at pH < 6.5.

The signal patterns of the seven histidine residues in a ^1^H‐^13^Cα correlation spectrum are shown in Figure 4a. A very specific pattern of Cα‐H cross peaks is obtained which appears only in the ^13^C chemical shift range between 54 and 56 ppm, as is typical for His. There are five intense cross peaks (green circles), two are less intense (yellow circles) at 5.3 ppm, and two intermediate cross peaks (light blue circles) that are a little further separated at 4.1 and 4.35 ppm. Cross sections of this spectrum are displayed in Figure S6. The S/N is significantly improved compared to that of the ^1^H‐^15^N correlation spectrum shown in Figure 3a, with values for the selectively labeled sample ranging from 3 for the weak peaks to above 8 for the stronger ones. Interestingly, the intensity of the small cross peaks around 5.3 and 5.4^1^H chemical shifts are in the range of 35%–50% of the largest (estimated by integration). It is striking that we observe nine well‐shaped cross peaks (i.e., with approximately elliptical line shapes, in contrast to the in general more irregular and nonuniform appearance of noise peaks) for seven His residues. The signal patterns of the Trp side chain cross peaks in ^1^H‐^15^N correlation spectra occur in the range between 9.7 and 11.2 ppm ^1^H chemical shifts and around 130 ppm ^15^N chemical shifts (Figure 4b–d), separated from the bulk of amide NH signals. This is confirmed by the CP‐based ^1^H‐^15^N correlation of the selectively Trp‐labeled sample (Figure 4c,d). Notably, the intensities of the cross peaks vary considerably. This is apparent from the cross sections shown in Figure S7 from spectra with different processing parameters, and integration of the three cross peaks indicated on top, where the small signal (Figure S7d,f) is around 25%–35% of the largest. The assignment of the side chain signal of W131 [16] may be transferred to the OM vesicle spectra, since this sidechain points toward the interior of the barrel and is thus not directly affected by changes in lipid environment. It is a measure of Trp signal intensity from a rigid part of the protein.

**FIGURE 4:**
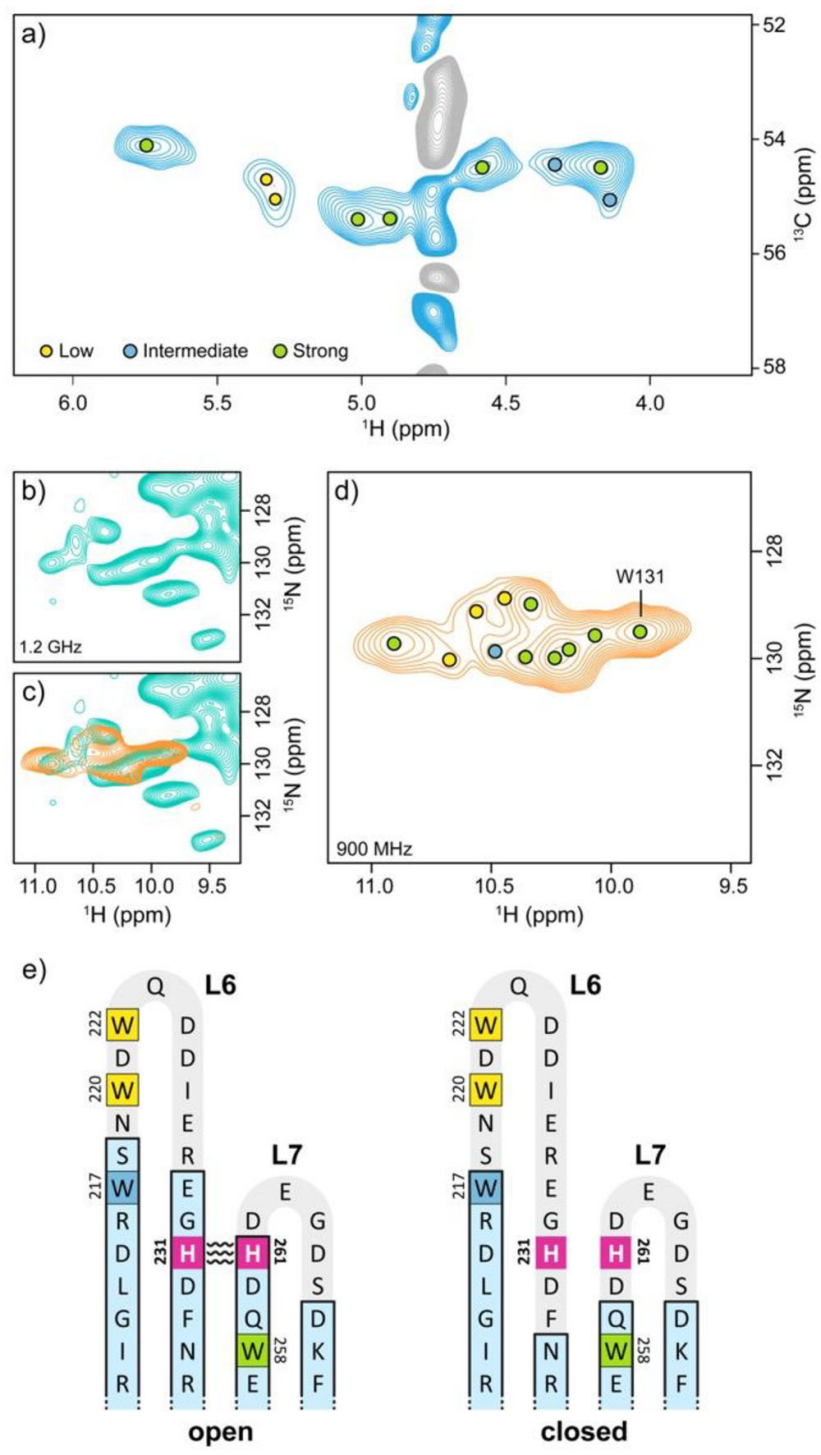
(a) C_α_H region of the hCH CP spectrum of [^13^C,^15^N‐His]‐labelled OmpG in OM vesicles. The filled circles indicate peak intensity. The artefact around 4.7 ppm arises from bulk water. (b) The Trp region of the hNH CP spectrum of U‐[^13^C,^15^N]‐labelled OmpG in OM vesicles. (c) Overlay of the spectrum in (b) with the hNH CP spectrum of [^13^C,^15^N‐Trp]‐labelled OmpG. d) hNH CP spectrum of [^13^C,^15^N‐Trp]‐labelled OmpG in OM vesicles now processed with an exponential multiplication in t_2_. A line broadening of 120 Hz was applied in F2. The filled circles indicate peak intensities, see scale in (a). (e) Schematic representation of loops 6 and 7 for both forms of OmpG, open (left) and closed (right). The background color of the Trp residues represents a tentative assignment of the intensities observed in the spectrum shown in Figure 4d to appropriate sequence positions using the same color code, see text. The black frames together with blue background indicate the extent of the β‐sheet as observed in the corresponding x‐ray structures.

For an interpretation of the His signal pattern, we assume that each of the large signals originates from a single distinct site, and that there are two pairs of histidine signals which are split. To interpret the small signals as offspring of the large does not look probable given their intensity of about 30% of the large ones. The observed pattern, however, fits to the structural basis that is available. In the set of x‐ray structures, five His residues (H7, H74, H97 H204, and H270) participate in β‐sheet formation in all x‐ray structures, irrespective of pH. Six x‐ray structures (PDB: 2IWW, 4CTD, 2F1C, 7Q5C, 2WVP, 2 × 9K) show H231 and H261 as members of loops 6 and 7, respectively, outside the rigid β‐sheet region. Only in the pH 7.9 x‐ray structure (PDB: 2IWV), all seven His residues are found in a regular β‐sheet environment due to the elongation of strands β12 and β13 at the expense of loop structure.

Our data corroborate a picture where the five most intense cross peaks originate from the residues located in the β‐sheet area that is conserved in all x‐ray structures. The two doubled cross peaks are likely due to the two His located within loops 6 (H231) and 7 (H261) of the NMR structures and of most x‐ray structures, including the x‐ray structure 2IWW (Figure 2). The fact that two pairs of small cross peaks occur at different places in the spectrum suggests that these residues can adopt two states that persist on a millisecond or larger timescale. The ^1^H chemical shifts of the pair around 5.3 ppm are characteristic of β‐structure, potentially in agreement with H231 and H261 located in an elongated sheet. In this picture, both H231 and H261 give rise to two distinct signals that appear in different spectral regions, one around 5.3 ppm and one around 4.1 – 4.35 ppm. The ^1^H chemical shifts of the cross peaks at 4.1 and 4.35 ppm do not specifically indicate any type of secondary structure, but they are in agreement with H231 and H261 potentially being part of a stretch of random structure or situated in mobile loops. In summary, the data suggest that the two loops 6 and 7 of OmpG in our OM vesicle preparation do not adopt a single rigid structure at pH 7.4; of the seven His residues, most likely H231 and H261 are present in at least two conformational states.

We may also interpret the pattern of Trp cross peaks in light of the existing structural basis. All x‐ray structures resolve seven Trp residues (W43, W65, W112, W118, W131, W217, W258) in the β‐barrel, some of which are directly at the strand ends (Figures 1b and 2a), with low B‐values indicating rigidity. W6 at the N‐terminus appears more flexible in some of the x‐ray structures, indicated by higher B‐values. Four Trp residues were seen to be mobile in the previously determined NMR structures, including one Trp at the end of β11 (W217), two in loop 6 (W220, W222), and one in β13 (W258) [14, 15]. Those are all located in the Loop 6 and 7 region (Figure 4e). Four Trp (W43, W112, W118, and W131) were assigned in our earlier study where they did not show signs of signal multiplication.

In the spectrum shown in Figure 4d, we identify seven strong cross peaks (Figure 4d, green circles), one intermediate (light blue circle), and three weak cross peaks (yellow circles), which includes one more peak than expected from the number of Trp residues. The integrals of the small cross peaks are around 30% of the large peaks, and we estimated the ‘intermediate’ signal (light blue circle) to be only slightly larger than the small ones. Thus, the cross‐peak pattern suggests that at least two Trp residues are rather flexible, and one has an intermediate degree of flexibility. Even without a sequence‐specific assignment of the Trp cross peaks it is fair to assume that all strong signals must arise from Trp residues in or close to the rigid barrel, as also suggested by the large integral of the W131 signal. A comparison of the pH 7.9 x‐ray structure (PDB: 2IWV), representing the open form, with the cross‐peak intensities observed in the CP‐based ^1^H‐^15^N correlation obtained at pH 7.4 reveals an interesting difference. In the crystallographic study loops 6 and 7 appear to have surprisingly low B‐values (Figure 2b) and thus all Trp residues are in a rigid environment, whereas the signal intensities in the NMR spectrum (Figure 4d) suggest intensity modulations by motion for some of them. Assigning the seven high‐intensity peaks to β‐barrel residues, the less intense peaks must arise either from the N‐terminal W6, which the two x‐ray structures show to be of intermediate flexibility, or from the two Trp residues in loop 6 (W220 and W222) that have the largest potential to be flexible. In Figure 4e, we have tentatively assigned the cross peak intensities to the four Trp residues situated in L6 and 7 using the same color code as in Figure 4d. In OM vesicles, the loop 6 Trp W220 and W222 are either surrounded by lipid A disaccharides or embedded in the LPS core oligosaccharide region (Figure 1a), which is an obvious difference to the situation in the crystal, where possible rigidification by crystal contacts comes into play. Since the loop 6 residues have low B‐factors in the pH 7.9 structure (PDB: 2IWV) of the open form, we also inspected the B‐factors in the x‐ray structure of the closed form (PDB: 2IWW) obtained at pH 5.6. In this structure, W220 and W222 have high B‐factors (Figure 2c), indicating high flexibility, and in agreement with the cross‐peak intensities in our NMR spectra. The rigidity of the loops observed in the x‐ray structure of the open form (Figure 2b) is thus likely due to stabilization by crystal contacts.

Taking our results together, we conclude that the oligosaccharide environment does not exclusively support a rigid loop structure as observed in the crystal at pH 7.9 (Figure 2b), but rather a more dynamic one that fits to a dynamics profile in the loop 6 region similar to the pH 5.6 structure (Figure 2c).

### 2.3 Accessibility of LPS‐Embedded Residues

The dynamics of loop residues may be further assessed by testing their solvent accessibility in fully deuterated samples from bacteria grown in 100% D_2_O, as is common in NMR [31]. Complete deuteration minimizes the ^1^H content at aliphatic sites and thus results in longer transverse relaxation times, that is, sharper signals, of protons that partition to solvent‐accessible sites when the deuterated protein is purified in an H_2_O‐containing buffer. It is generally expected that strongly hydrogen‐bonded or membrane‐integrated N‐D groups exchange very slowly, in a timeframe of weeks to months, and therefore the proton signals of such groups will be absent [32]. We take advantage of these vastly different exchange rates for analytical purposes by recording two types of NMR experiments, one employing CP, the other employing an INEPT excitation scheme [25]. The CP‐ (Figure 5a) and INEPT‐based (Figure 5b) ^1^H‐^15^N correlations of a perdeuterated and back‐exchanged sample are clearly complementary. In the range between 9.6 to 11 ppm, where cross peaks of β‐sheet residues and Trp side chain ^1^H‐^15^N cross peaks are expected, the CP‐based spectrum shows only the cross peak of the W131 side chain ^1^H‐^15^N group. We attribute the exclusive appearance of W131 NH to its location in the interior of the β‐barrel where it can exchange easily while still being firmly embedded in its β‐sheet environment. The W131 side chain NH group is thus not involved in strong hydrogen bonding. The INEPT ^1^H‐^15^N correlation shows a few cross peaks at chemical shifts higher than 9.6 ppm, with now three Trp side chain cross peaks. The two signals marked by arrows in Figure 3b do not appear in the CP spectrum but only in the INEPT‐based ^1^H‐^15^N correlation. Their presence indicates, at the same time, NH group accessibility enabling D to H exchange and rapid motion as a prerequisite of effective INEPT‐based magnetization transfer. Not surprisingly, these two signals correspond to weak CP signals in Figure 4b. Furthermore, they are observed only for OmpG in the OM but not in reconstituted bilayers. We conclude that these Trp residues are the most dynamic, and likely the closest to the membrane surface. The obvious candidates are W220 and W222. The absence of most other Trp signals suggests hydrogen‐bonding interactions of their side chain moieties with oligosaccharide groups, or protection from exchange in the lipid phase. The W131 cross peak occurs in both spectra, demonstrating a rigid environment in which MAS is most effective, resulting in narrow proton and nitrogen lines so that INEPT transfer is also possible [25].

**FIGURE 5:**
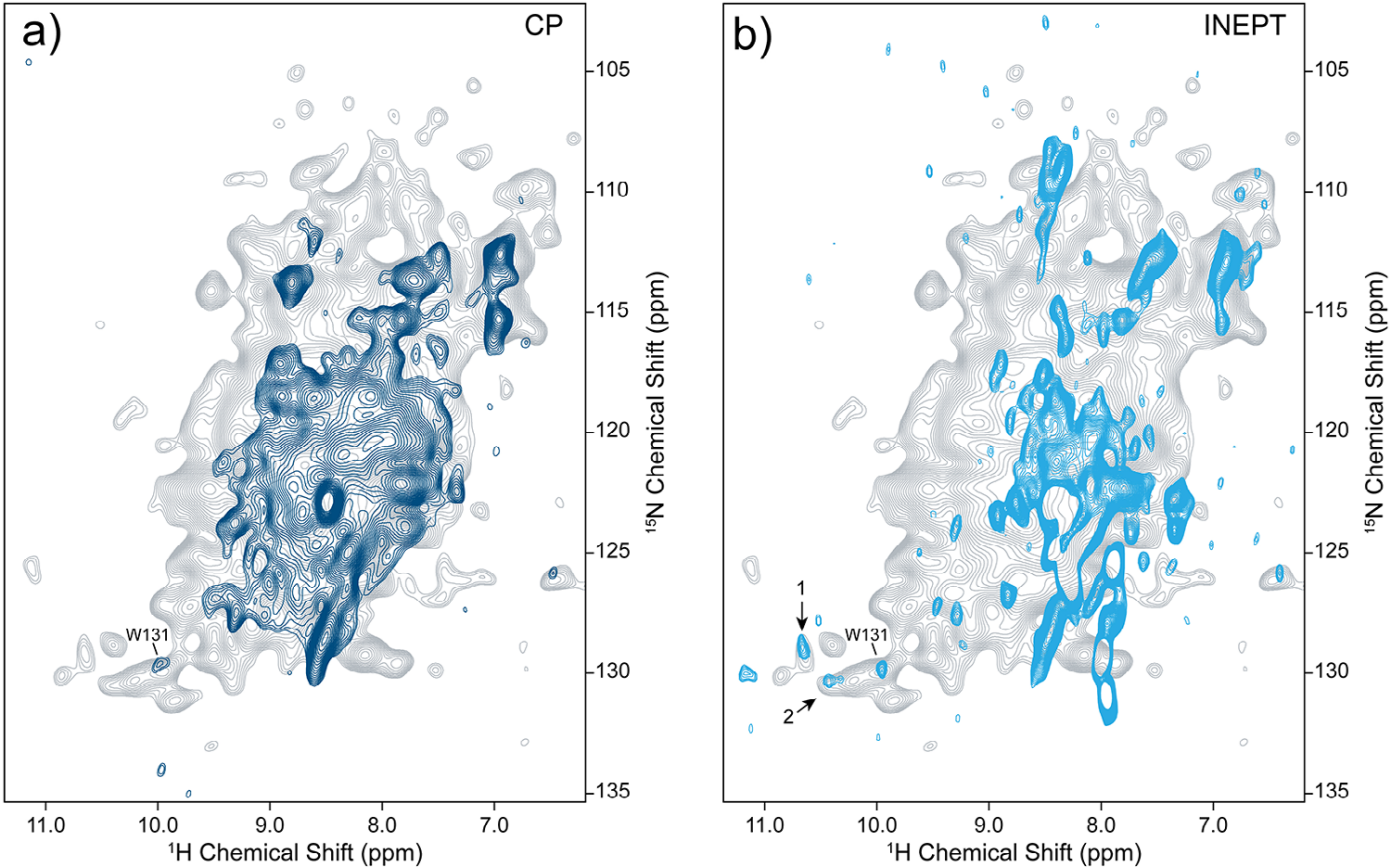
(a) ^1^H‐^15^N correlation of [^2^H, ^15^N]‐OmpG in OM vesicles recorded by applying cross polarization (CP, dark blue) at 60 kHz MAS and 600 MHz ^1^H frequency, overlaid on the [^13^C,^15^N]‐labelled OmpG spectrum (grey, see Figure 3a). b) INEPT‐based ^1^H‐^15^N HSQC spectrum (light blue) recorded at 100 kHz MAS and 900 MHz ^1^H frequency. The reference spectrum of Figure 3a is shown in the background (grey). For arrows 1 and 2, see text.

## 3 Conclusions

Membrane proteins present multiple challenges for structural biology. Their structure and its preservation are often affected by experimental conditions during sample preparation, in particular the choice of membrane mimetics, which can be various lipids, detergents, or amphipols [33]. This applies in particular to loop structures that extend from the lipid bilayer at the membrane surface. The loops are more sensitive to changes in the chemical environment than the hydrophobic core regions with their relatively robust, lipid‐embedded β‐sheet or alpha helix structures. This observation prompted a number of recent investigations that made use of OM vesicles [3, 34, 35, 36, 37], including a study of histidine protonation within in the extracellular loops of the protein PagC, which provided a close‐to‐native picture [37].

The methods used for structure determination, each with their own technical constraints and opportunities, can also affect the outcome. In this respect, OmpG is an instructive example, as x‐ray crystallography and NMR highlight complementary aspects of its structure, reflecting the experimental requirements of both methods. The OmpG loops are thought to mediate the opening and closing of the OmpG pore. We therefore aimed at investigating structural states closer to the structure in the native membrane, and the associated dynamics, by employing OM vesicles and ssNMR. Although a high‐resolution structure was not obtained, a number of characteristic features became apparent. (i) We find for two Histine residues that each is represented by two cross peaks. Although a potential reason for this duplication could be membrane heterogeneity, we assume that these are H231 and H261 since the other His residues are located in the conserved ß‐sheet and no His cross peak duplication was observed for the previously assigned H7, H74 and H205. The ^13^C chemical shift pattern of the 7 histidine residues (Figure 4d) all confirm beta‐ or random coil structure (ppm range between 54.0 and 55.5 ppm). The proton chemical shifts of the small peaks around 5.3 ppm are consistent with a ß‐sheet structure and would account for H231 and H261 within the antiparallel strands observed by x‐ray crystallography of the open form. (ii) Transient interactions with the oligosaccharide layer result in slower motions than observed in previous solution and solid‐state NMR projects as exemplified by the substantial CP observed in our experiments, yet the oligosaccharide layer does not appear to have a large ordering effect. (iii) Although the variation in strength of the Trp NεH signals in CP experiments indicate considerable mobility of the Trp sidechains, their accessibility to the medium is limited, suggesting transient hydrogen bonding within the oligosaccharide network.

Our study applies advanced NMR technology to the investigation of membrane protein structure and function. With OmpG embedded in the oligosaccharide layer of the OM vesicles at pH 7.4, we now observe an extended set of Trp side chain ^1^H‐^15^N signals, and a set of His backbone cross peaks that is exceeding the number expected for seven His, suggesting two conformations for the loop 6/β12 and loop7/β13 His residues (Figure 4e). We detected the additional Trp and His signals at proton frequencies of 600, 900 and 1200 MHz (Figure S1). Recording spectra in a quasi‐native OM environment thus can be even more informative than utilizing the highest available field strength. In an NMR study of OmpG in dodecylphosphocholine micelles [15] the signals of loop 6 were not identified or detected. According to the authors of this previous study, this was most likely the case because of line broadening due to chemical exchange between the open and closed forms. The signal pattern obtained in this study fits to such a hypothesis. This investigation highlights the importance of revisiting outer membrane protein (Omp) structures in mechanistic studies by investigating them in a quasi‐native environment, for which solid‐state NMR offers unique opportunities.

## Conflicts of Interest

The authors declare no conflicts of interest.

## Supporting information



The authors have cited additional references within the Supporting Information [38, 39, 40, 41, 42, 43, 44, 45, 46, 47].
**Supporting File 1**: chem71575‐sup‐0001‐SuppMat.pdf.

## Data Availability

The data that support the findings of this study are available on request from the corresponding author. The data are not publicly available due to privacy or ethical restrictions.
