# Supplementary material for: Structure and Dynamics of Porin Loops in Asymmetric Outer Membranes of E. coli
Source: Chemistry. 2026 Aug 14;32(41):e71575. doi: 10.1002/chem.71575 (PMC13729952; doi:10.1002/chem.71575)
Supplement: Supplementary file 1 [file CHEM-32-e71575-s001.pdf]

WILEY-VCH

Supporting Information

Structure and dynamics of porin loops in asymmetric outer membranes of *E. coli*

*Morgane Adélaïde, Jayasubba Reddy Yarava, Florian Lindemann, Massilia Abbas, Lisa Gerland, Matthias Hiller, Barth van Rossum, Dirk Linke, Alexander B. Barnes, Edward P. Saliba, Özkan Yildiz, Werner Kühlbrandt & Hartmut Oschkinat**

Table of content

Abbreviations S2
Figure S1 .... S3
Figure S2 .... S4
Figure S3 .... S5
Figure S4 .... S6
Figure S5 .... S7
Figure S6 .... S8
Figure S7 .... S9
Experimental Methods S10
Table S1 .... S11
Solid-State NMR Spectroscopy S12
Table S2 .... S13

Abbreviations

CP: Cross Polarization
DPC: Dodecyl phosphocholine
EDTA: Ethylenediaminetetraacetic acid
HEPES: 2-[4-(2-Hydroxyethyl)piperazin-1-yl]ethane-1-sulfonic acid
KDO: 3-deoxy-D-manno-oct-2-ulosonic acid
INEPT: Insensitive Nuclei Enhanced by Polarization Transfer
IPTG: Isopropyl ß-D-1-thiogalactopyranoside
LamB: Lambda receptor B
LPS: Lipopolysaccharides
MAS: Magic Angle Spinning
OmpG: Outer membrane protein G

WILEY-VCH

Figure S1. Comparison of 2D ^{1}H-^{15}N correlation spectra of [^{13}C,^{15}N]-OmpG in OM vesicles at different field strength, focusing on the region with cross-peaks due to Trp side chain ^{1}H-^{15}N correlations. The spectra of U-[^{13}C,^{15}N]-OmpG in OM recorded at ^{1}H frequencies of 1200 MHz (a) and 900 MHz (b) result from the same sample. The spectrum of [^{13}C,^{15}N-Trp]-OmpG in OM vesicles recorded at a ^{1}H frequency of 900 MHz is shown in (c). The spectra were recorded using probes for 0.7mm diameter rotors, operating at MAS rates of 100 kHz, and at a sample temperature of around 30 °C.

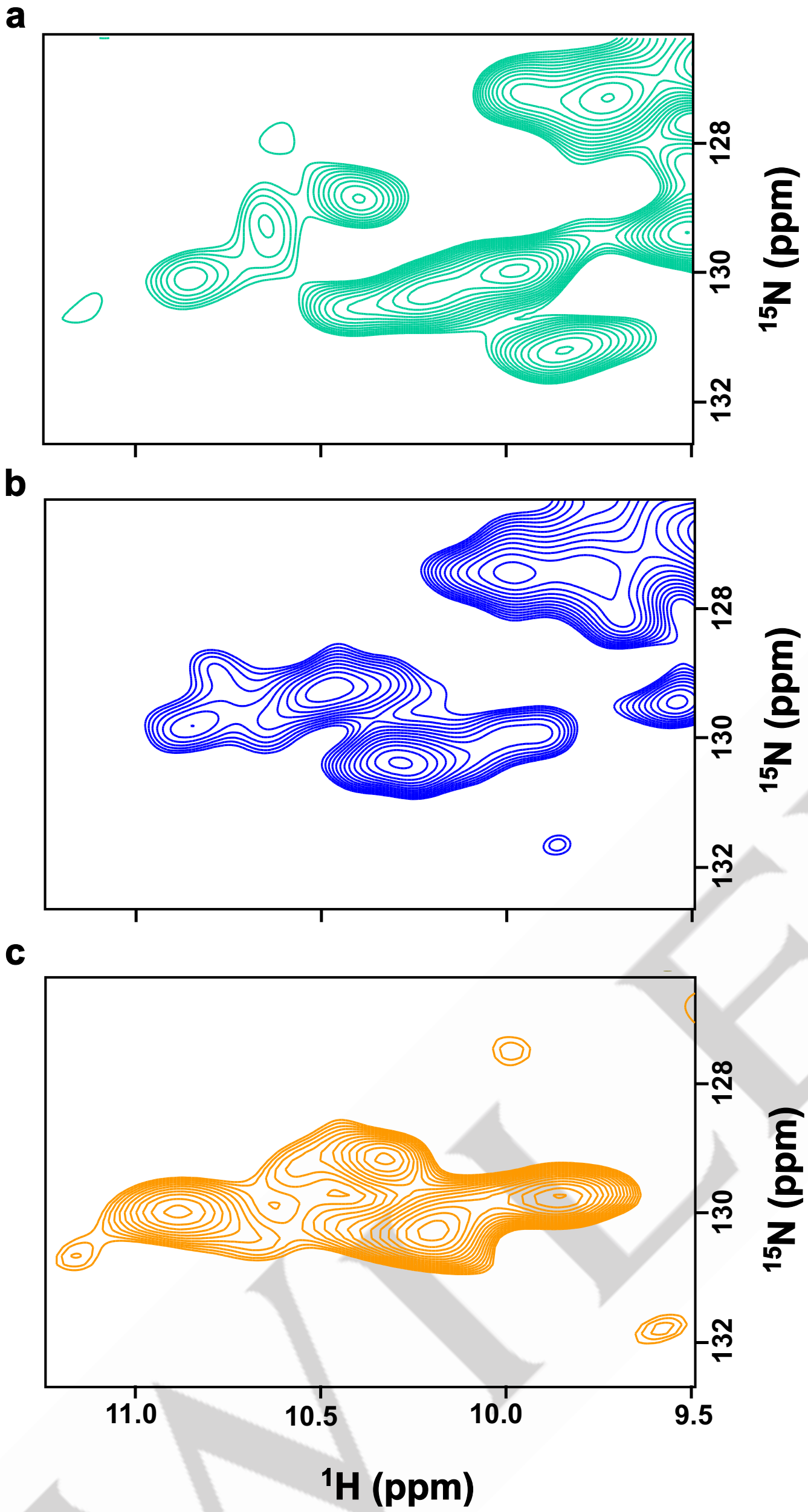

Figure S2. Comparison of 2D ^{1}H-^{15}N correlation spectra of [^{13}C,^{15}N]-OmpG in OM at 1200 MHz ^{1}H frequency (turquoise) with [^{13}C,^{15}N]-OmpG in OM at 900 MHz ^{1}H frequency (dark blue), using exactly the same sample. The spectra were recorded using a probe for rotors with a diameter of 0.7 mm, operating at a MAS rate of 100 kHz, and at a sample temperature of around 30 °C. The 900 MHz spectrum shows artifacts from baseline correction in F1 (^{15}N dimension).

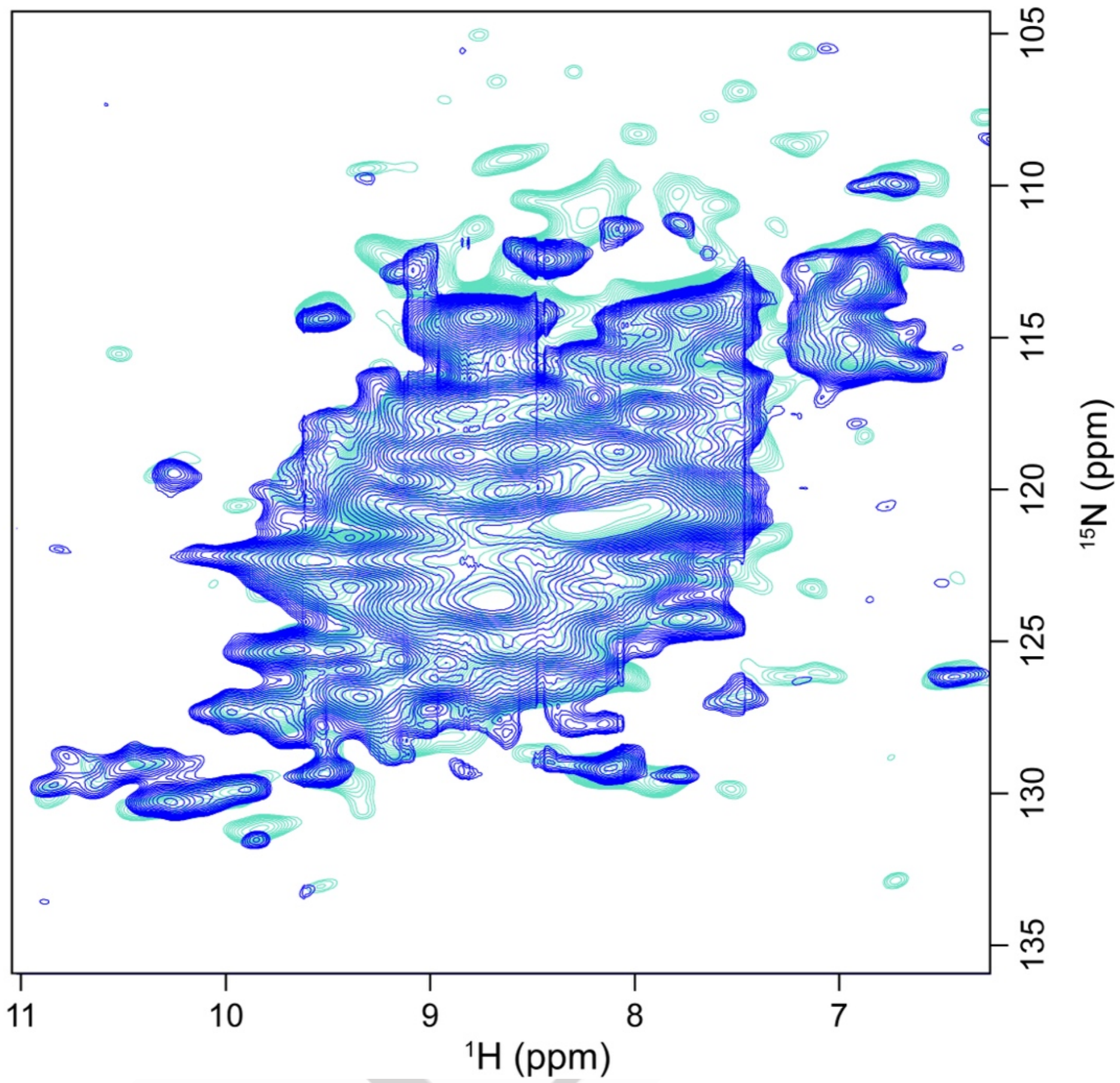

Figure S3. 1D ^{1}H NMR spectra of the ^{13}C, ^{15}N labelled sample when fresh, measured at 1.2 GHz (a) and after weeks of measurement and storage time, recorded at 900 MHz (b). In a) the ratio of peak1 (water peak) and peak2 (LPS sugar signals) is 21, in b) it is 19.

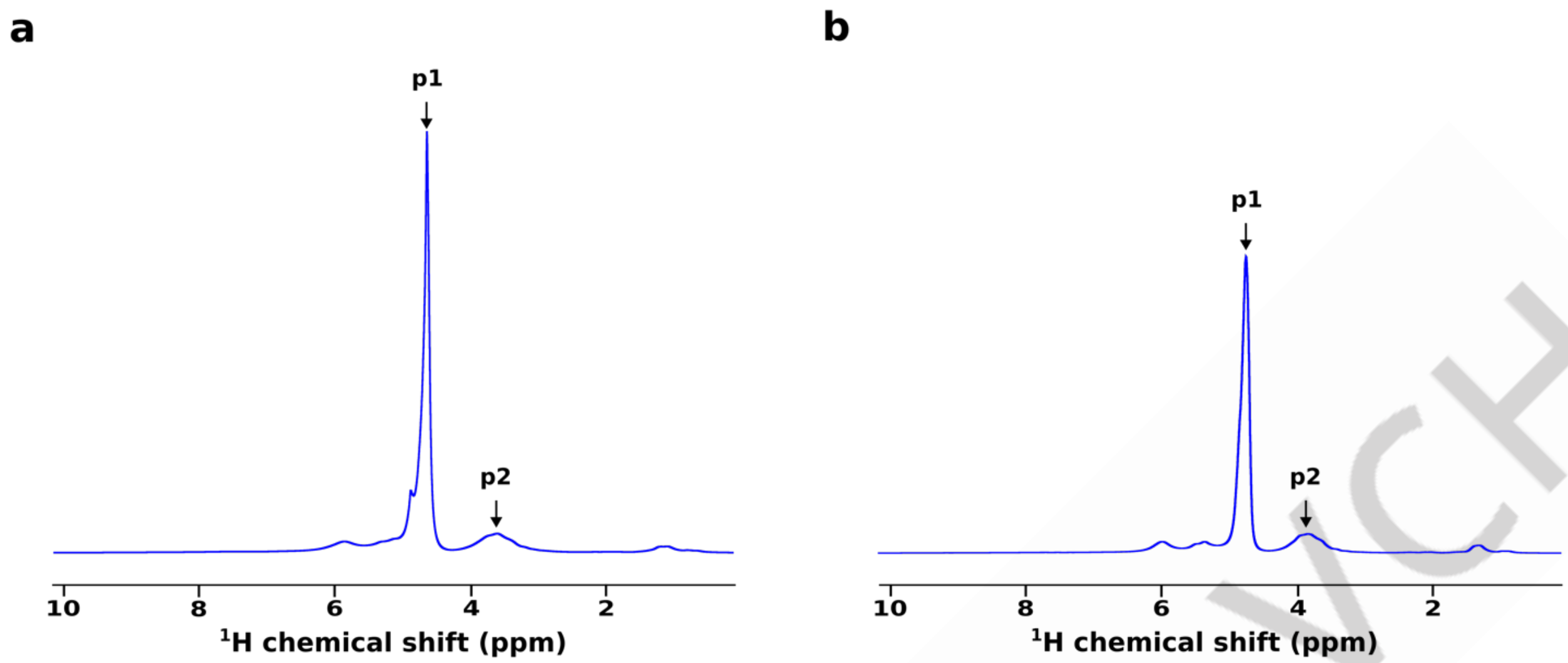

WILEY-VCH

Figure S4. F2-Traces of the indicated cross peaks in Figure 3a. The horizontal arrows in b-g indicate the spectral region that served for quantification of the noise using the 'sino' command in the Bruker program TopSpin.

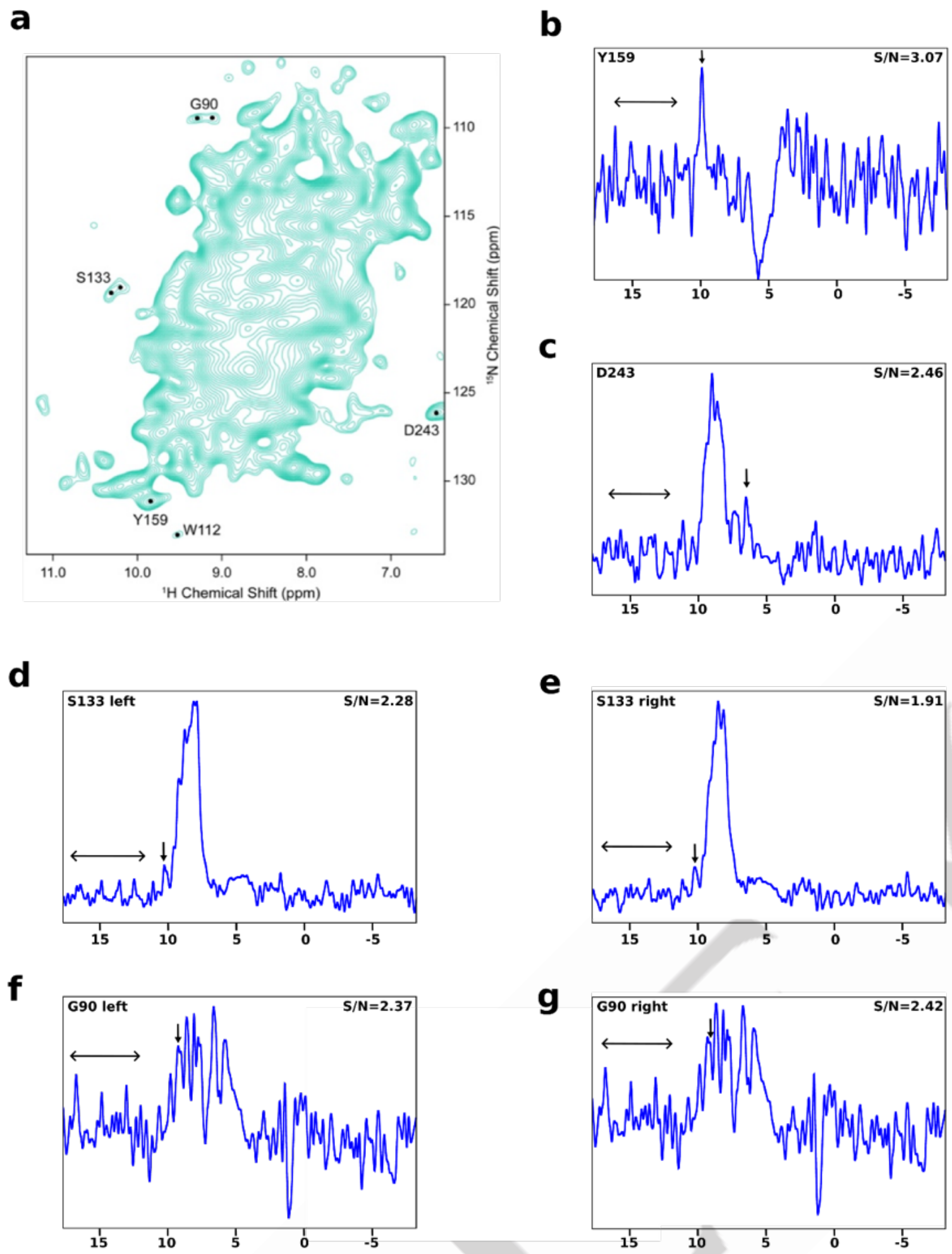

WILEY - VCH

Figure S5. Overview plot of the ^{1}H-^{13}C correlation spectrum of 13C, 15N His-labeled OmpG in OM (a) and an overview plot of the ^{1}H-^{15}N correlation spectrum of 13C, 15N Trp-labeled OmpG in OM (b).

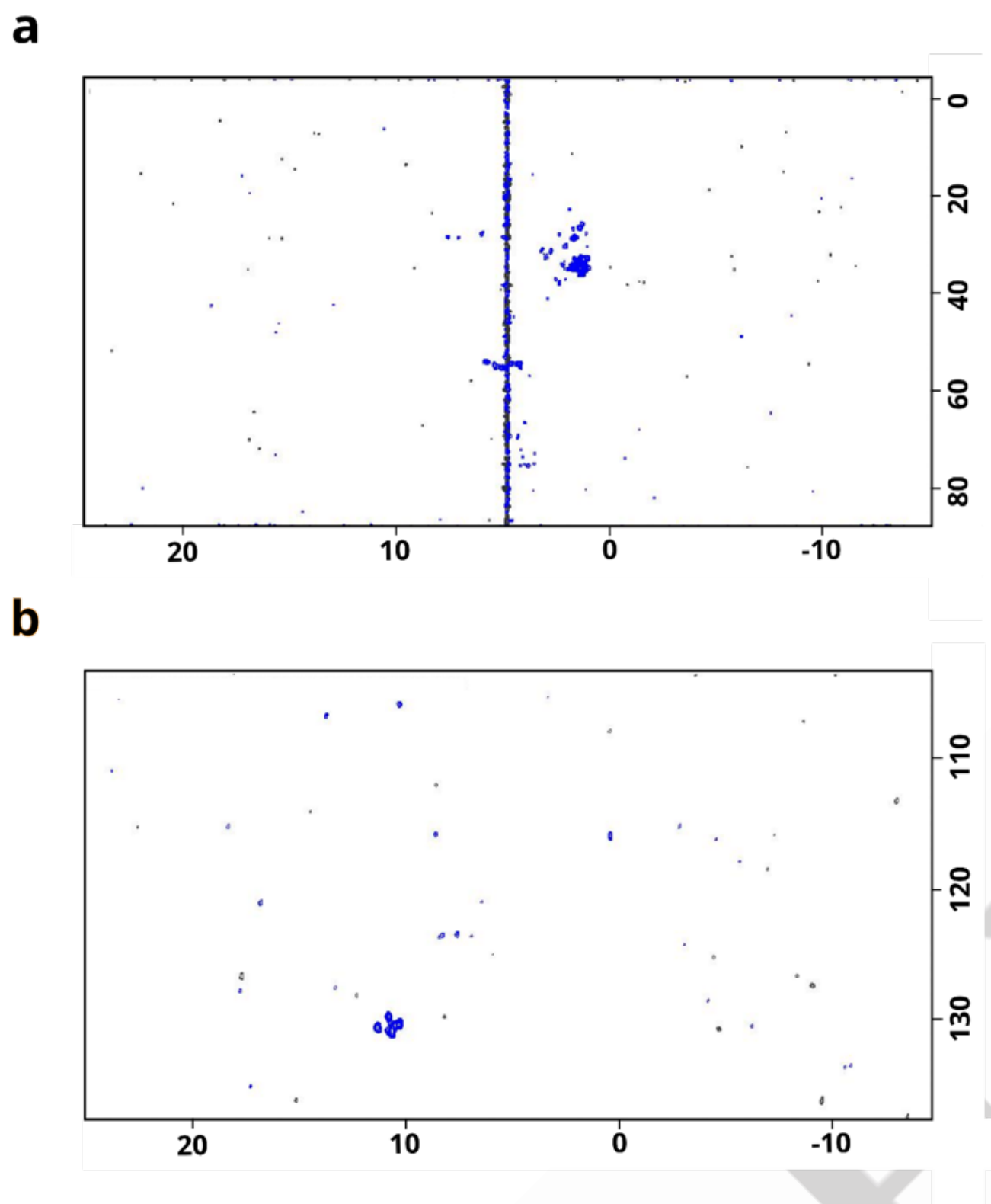

WILEY-VCH

Figure S6. Spectral region of the ^{1}H-^{13}C correlation spectrum of 13C, 15N His-labeled OmpG in OM covering Ha-Ca cross peaks (a) and F2 traces of 4K data points taken at the indicated points. The horizontal arrows in b-f indicate the spectral region that served for quantification of the noise using the 'sino' command in the Bruker program TopSpin.

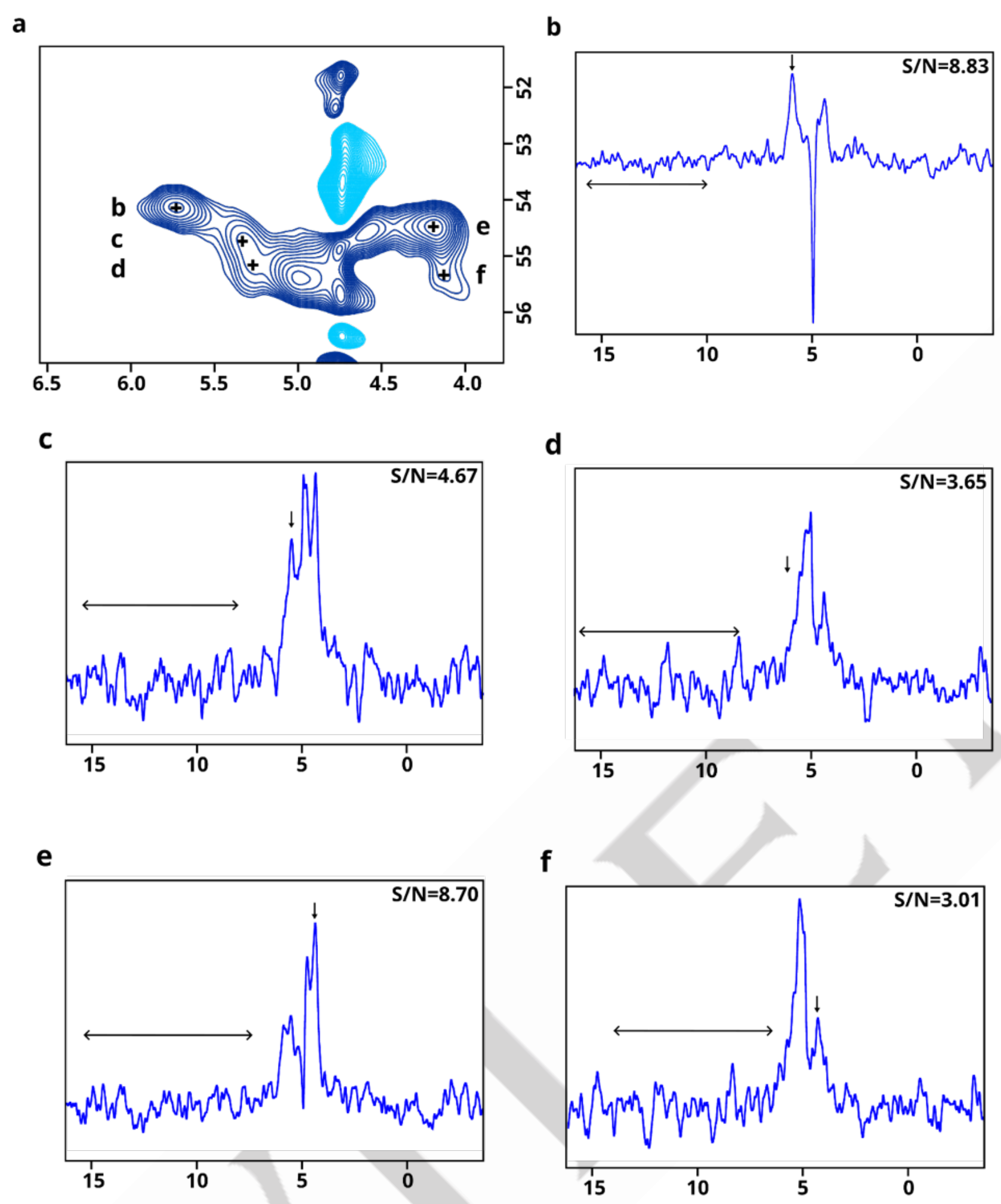

Figure S7. Spectral region of the spectrum in Figure S5b exposing the side chain 1H-15N correlations of the Trp residues. The points were the F2-cross sections were taken are indicated by small letters. The two columns represent different processing (Gaussian multiplication in the first column, exponential multiplication with a line broadening of 120 Hz in the second). 4K data points were used in F2. The horizontal arrows in b-d and f-h indicate the spectral region that served for quantification of the noise using the 'sino' command in the Bruker program TopSpin.

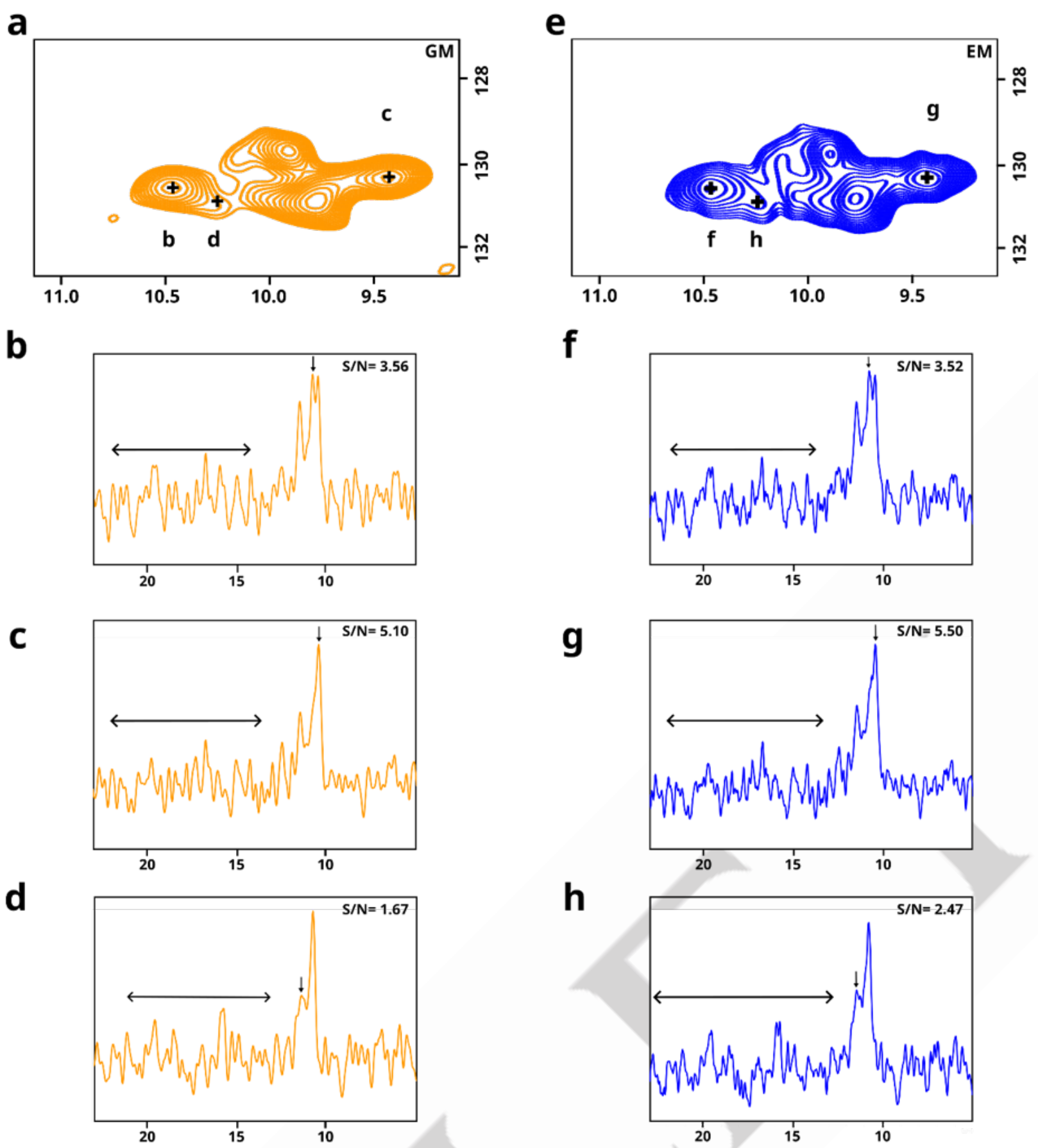

WILEY-VCH

Experimental Methods

Protein Preparation

Uniformly [^{13}C,^{15}N]-labelled OmpG was expressed into the membrane of the *E.coli BL21 ΔABCF* whereby ΔABCF denotes the deletions of four genes encoding abundant β-barrel proteins in the OM (*ompA, ompC, ompF,* and *lamB*) as described[12,23]. Cells were transformed with the vector pET-26b-ssA-OmpG(22-301), encoding a recombinant protein of 280 residues, plus an N-terminal Met. ssA denotes the presence of the OmpA signal sequence. A single colony of transformed BL21 ΔABCF cells was resuspended in 4 mL LB medium and grown at 37 °C, 170 rpm. At a cell density of OD_{600} = 1, the cells were diluted into 50 mL of LB supplemented with ^{12}C-glucose (0.2%) and 50 µg/mL kanamycin, then maintained over night at 25 °C, 170 rpm. An appropriate amount of cell mass was collected by centrifugation (3000g, 10 min) and used to inoculate a 1 L culture of M9 minimal medium containing ^{15}N-NH_4Cl (0.4 g/L), U-[^{13}C]-glucose (2 g/L), mineral salts and kanamycin at 50 µg/mL (Table S1) so that a final OD_{600} of 0.2 was reached. The culture was maintained at 30 °C and 170 rpm. When the culture reached a cell density of OD_{600} = 0.7**,** expression of OmpG was induced by adding 0.05 mM of isopropyl ß-D-1-thiogalactopyranoside (IPTG). Expression took place overnight at 20 °C**.** Cells were harvested by centrifugation (5000 g, 15 min, 4 °C), washed in 0.9% NaCl solution and stored at – 80°C.

To produce [^{2}H,^{13}C,^{15}N]-labelled OmpG, an appropriate number of cells was resuspended in 200 mL of M9/D_2O medium enriched in ^{15}N-NH_4Cl (0.4g/L), U-[^{2}H,^{13}C]-glucose (2 g/L), and supplemented with mineral salts and kanamycin at 50 µg/mL (Table S1). The culture was maintained overnight at 30 °C, 150 rpm, then all of it was used to start a 1L culture of M9/D_2O medium with the same composition as above and maintained at 160 rpm, 34°C for 1h at 30 °C. At a cell density of OD_{600} = 0.6, the expression of OmpG was induced by adding 0.05 mM IPTG. Expression was maintained over night at 20 °C, 160 rpm and cells were harvested by centrifugation (5000 g, 15 min, 4 °C), washed in 0.9 % NaCl solution and stored at -80°C.

[^{13}C,^{15}N]-histidine-labelled OmpG or [^{13}C,^{15}N]-tryptophan-labelled OmpG were prepared as the uniformly [^{13}C, ^{15}N] labelled OmpG sample except that after collecting an appropriate amount of cells by centrifugation, cells were used to inoculate a 1L culture of minimal medium M9/H_2O containing ^{14}N-NH_4Cl (0.4g/L), U-[^{12}C]-glucose (2 g/L) and 100 mg/L [^{13}C,^{15}N]-tryptophan or 100 mg/L [^{13}C,^{15}N]-histidine respectively, supplemented with mineral salts and kanamycin at 50 µg/mL. Everything else being similar as for the uniformly [^{13}C,^{15}N]-labelled sample.

Outer membrane preparation: Cell pellets were taken up in lysis buffer A (10 mM 2-[4-(2-Hydroxyethyl)piperazin-1-yl]ethane-1-sulfonic acid (HEPES), 2 mM $MgSO_4$, pH 7.4) in the presence of 25 U/mL DNase and 1x Complete EDTA-free at a final cell density of 10 mL of buffer/g of cells. Cells were lysed by five passages at 13,000 psi through a high-pressure homogenizer (LM10, Microfluidics). Non-broken cells were then removed by centrifugation at 4000 g for 20 min and the total membrane fraction was collected by ultracentrifugation for 1 h at 100,000 g (Beckman 45 Ti). The inner membrane was solubilized by incubation in 1 mL of solubilization buffer (10mM HEPES pH 7.4 with 1% N-laurylsarcosine) per 50 mL of cells at OD_{600} of 1.5 for 45 min under gentle rocking at RT followed by an ultracentrifugation for 1 h at 100,000 g (Beckman 70.1 Ti) allowing outer membrane collection. The outer membrane pellet was washed 4 times in 1 mL of buffer B (10 mM HEPES pH 7.4), including a centrifugation step for 30 minutes at 100,000 g (Beckman TLA55) to remove detergent, and sample quality was checked by 12% SDS-PAGE. The protein-containing vesicles were sedimented into a Bruker 0.7 mm rotor by centrifugation for 48 h using a Beckmann SW 40 Ti rotor at 85073 g and 4°C, making use of a home build filling tool.[36]

Table S1. Sample preparation of labelled-OmpG in OM. [a]Approximation of the total protein amount using the Direct Detect infrared quantification system.

Labelling	$^{13}C,^{15}N$	$^{13}C,^{15}N$-Trp	$^{13}C,^{15}N$-His	$^{2}H,^{13}C,^{15}N$
^{13}C Glucose [g/L]	2.8	2.0	2.0	2.0
^{15}N NH_4Cl [g/L]	0.7	0.5	0.5	0.5
CN-amino-acid [mg/L]	-	100 mg/L Trp[a]	100 mg/L His[a]	-
D_2O/ SilantesDCN	-	-	-	100% / 0%
IPTG [mM]	0.05	0.05	0.05	0.05
Temp/ Time	18°C/ 18h	18°C/ 18h	18°C/ 18h	20°C/ 18h
Volume [L]	0.4	0.5		1.0
Yield biomass [g]	2.6	4.2		2.0
HD-back exchange	-	-		N/A
Solubilization ratio	10 mL per gram of cells	10 mL per gram of cells		5 mL per gram of cells
Temp / Time	RT / 30min	RT / 30min		RT / 30min
Washing steps	6 x 2mL	6 x 2mL		6 x 2mL
Yield OM [µL]	120-150 (5%[a])	150-180 (5%[a])	150 (8%[a])	N/A
Additive	EDTA, NaN_3 Pefabloc	-	EDTA-free, NaN_3	-
Sample preparation #	2351	2341	2381	2157
Figure	Fig 3a, S2	Fig 4c, 4d	Fig 4a	Fig 5a, 5b

WILEY-VCH

Solid-State NMR Spectroscopy

Two-dimensional (2D), CP-based ^{1}H-^{15}N correlation spectra (hNH)[37] were recorded on U-[^{13}C,^{15}N]-OmpG, [^{13}C,^{15}N-Trp]-OmpG and [^{13}C,^{15}N-His]-OmpG, all embedded in outer membrane vesicles. Experiments were recorded on standard-bore 1200 MHz Bruker Avance NEO spectrometers using a Bruker 0.7 mm triple-resonance probe, and further on a 900 MHz Bruker Avance NEO spectrometer or a 600 MHz Bruker Avance NEO spectrometer. In the case of measurements at 1200 MHz, the variable temperature was controlled using a Bruker cooling unit and adjusted to 240 K in order to reach a sample temperature of ~300 K. Temperature calibration was achieved *via* a water sample in D_2O with DSS.[38] The magic angle has been adjusted "on sample" by measurement of T2' proton transverse relaxation times and adjustment of the magic angle until the highest signal was obtained.[39]

^{1}H decoupling was achieved with swept-low power Two-Pulse Phase Modulation (TPPM) during nitrogen or carbon chemical shift monitoring.[40] The Multiple Intense Solvent Suppression Intended for Sensitive Spectroscopic Investigation of protonated proteins, the MISSISSIPI sequence, applied without gradients, was used for water suppression.[41]

All spectra were acquired using time-proportional phase incrementation (States-TPPI) in the direct dimension to obtain pure phase line shapes and phase discrimination.[42] Spectra recorded longer than 24 h were acquired in blocks and summed up afterwards. Between each block, the field was adjusted to compensate for non-linearity of the drift. Further information regarding data acquisition parameters for all 2D spectra can be found in Table S1-S2.

Data were processed using TopSpin 4.3.0 (Bruker TopSpin). The 2D spectra were usually processed using a shifted squared sine-bell apodization function (QSIN) in t_1 (SSB=2 or 3) and a GM apodization function in t_2 with typical values of LB=-50 and GB ranging from 0.005 to 0.013. Further processing parameters are in Table 3. Chemical shift and linewidth analysis was performed in CcpNmr Analysis 3.2.0.[43,44,45]

In order to roughly estimate the intensities of cross peaks in the two-dimensional spectra, they have been integrated using the integration mode of TopSpin.

Signal-to-noise calculations were performed with the 'sino' command in TopSpin using the parameters displayed in the relevant spectra of Figures S4, S6 and S7.

WILEY-VCH

Table S2. Experiment table for solid-state NMR acquisition used for [^{13}C,^{15}N]-, [^{13}C,^{15}N-Trp]-, [^{13}C,^{15}N-His]- or [^{2}H,^{13}C,^{15}N]-OmpG in OM. Regarding the shape of the pulse, a tangential profile was used, or a ^{1}H or ^{15}N linear ramp % (centered in kHz). ([a]) In this case Waltz64 was used.

2D Experiment	hNH CP			hCH CP	hNH CP	hNH INEPT
Labelling	CN	CN	CN-Trp	CN-His	DCN	DCN
Figure	Fig 3a	Fig S2	Fig 4c, 4d	Fig 4a	Fig 5a	Fig 5b
^{1}H Field / MHz	1200	900	900	900	600	600
Rotor diameter / mm	0.7	0.7	0.7	0.7	1.3	1.3
MAS frequency / kHz	100	100	100	95	60	60
Number of scans	240	668	576	64	128	128
Experiment time	17h3	65h44	42h5	16h12	11h34	30h31
t_1 increment	152	188	80	512	512	512
Sweep width (t_1) / ppm	68	69	34	21	197	197
Acquisition time (t_1) / ms	9.1	15	12.8	12.3	21.3	21.3
t_2 increment	2048	2048	1536	1536	2048	2048
Sweep width (t_2) / ppm	26	40	40	40	40	40
Acquisition time (t_2) / ms	32.8	28.7	21.5	21.5	42.6	42.6
^{1}H decoupling / kHz	8	9	9	8	11	15
^{15}N dec (WALTZ16) / kHz	2([a])	5	10	5	10	10
^{13}C dec (WALTZ16) / kHz	-	5	5	12	10	9
MISSISSIPPI /ms	44	110	110	120	100	100
MISSISSIPPI / kHz	25	15	14	14	14	15
Interscan delay /s	1.6	1.2	1	1.6	1.5	1.5
**Transfer 1**	**HN CP**	**HN CP**	**HN CP**	**HC CP**	**HN CP**	**INEPT**
^{1}H field / kHz	134	78	70	72	48	-
^{15}N or ^{13}C field / kHz	43	29	34	27	18.7	-
Shape	^{1}H linear ramp % (center in kHz)				Tan ^{1}H	-
	30% (114)	20% (70)	20%	40% (58)	-	-
CP contact time / ms	1	1.2	1.2	0.3	1.6	-
INEPT delay (1/2 J) / ms	-	-	-	-	-	3.6
**Transfer 2**	**NH CP**	**NH CP**	**NH CP**	**CH CP**	**NH CP**	**INEPT**
^{1}H field /kHz	134 (11.5)	78 (1)	70 (1)	72 (1.1)	50 (1.2)	-
^{15}N or ^{13}C field/kHz (power/W)	43 (16.4)	24 (10)	27 (14)	27 (2.5)	16.6 (2.2)	-
Shape	^{1}H linear ramp X% (center in kHz)				Tan ^{1}H	-
	30% (114)	20% (70)	^{15}N 40% (22)	40 % (58)	-	-
CP contact time / ms	1	0.8	0.8	0.4	1.2	-
INEPT delay (1/2 J) / ms	-	-	-	-	-	3.6
Carrier ^{15}N/ ^{13}C	117.5	120	120	- /40	118/ 100	115.5
Carrier ^{1}H	4.8	-	5.3	5.5	4.0	4.0

WILEY-VCH
